# A peculiar cave species of *Tomocerus* (Collembola, Tomoceridae, Tomocerinae) from Vietnam, with a discussion of the postantennal organ and prelabral chaetae in Tomocerinae

**DOI:** 10.3897/zookeys.408.7030

**Published:** 2014-05-12

**Authors:** Daoyuan Yu, Feng Zhang, Louis Deharveng

**Affiliations:** 1 School of Life Sciences, Nanjing University, Nanjing 210093, China; 2 Department of Entomology, College of Plant Protection, Nanjing Agricultural University, Nanjing 210095, China; 3 Muséum National d’Histoire Naturelle, UMR7205 CNRS/MNHN, CP50, 45 rue Buffon, 75005 Paris, France

**Keywords:** New species, Southeast Asia, taxonomy, *Tomocerus folsomi*, *Tomocerus ocreatus*, *Tomolonus*

## Abstract

The first *Tomocerus* species with a postantennal organ (PAO) in the adult stage is described from Vietnam. *Tomocerus postantennalis* **sp. n.** differs from the other PAO-possessing tomocerid, *Tomolonus reductus* Mills, 1948, mainly in the morphology of PAO, the number of ocelli, the number of chaetae in trochantero-femoral organ and several features of the furca. The new species is placed in *Tomocerus* because of the presence of a toothlet on the outer basal mucronal tooth and the absence of the diagnostic character states of *Plutomurus* Yosii, 1956 and *Aphaenomurus* Yosii, 1956. Besides the presence of PAO, the new species is peculiar in having six prelabral chaetae, instead of four as in other *Tomocerus* species. The new species is similar to *Tomocerus folsomi* Denis, 1929 and *Tomocerus ocreatus* Denis, 1948 in the type of dental spines but different from them in the body colour, the relative length of antennae to body, the number of unguis inner teeth and the number of mucronal intermediate teeth.

## Introduction

In Collembola, PAO is a paired organ located dorsally on the head, behind the antennae. It is probably a sensory organ of smell, humidity or temperature (Altner and Thies 1976). In some group, e.g. Onychiuridae and Neanuridae, PAO is morphologically diversified and highly important for taxonomy (Hopkins 1997). In contrast, most Entomobryomorpha have this organ poorly developed (e.g. Isotomidae) or absent (e.g. most Entomobryidae, Tomoceridae, Paronellidae and Cyphoderidae).

PAO in Tomocerinae was firstly recorded by Mills (1948) in a new genus and species, *Tomolonus reductus* Mills, 1948. Goto (1956) showed that PAO is present in the first instar of *Pogonognathellus longicornis* (Müller 1776) but disappears in subsequent instars. Christiansen (1964) claimed this organ did not always appear in all specimens of *Tomolonus reductus* and accordingly rejected the generic state of *Tomolonus*, but later studies (Yosii 1967, Rusek 1977) supported Mills’ original position.

Until now, the only known species of Tomocerinae with PAO in the mature stage was *Tomolonus reductus*, from North America. The purpose of this paper is to describe a second species of this subfamily with well developed PAO, based on material from Vietnam.

## Materials and methods

All type specimens were collected in caves with aspirators. After being photographed with a Jenoptik ProgRes C10+ camera mounted on a Leica MZ 16 stereomicroscope, specimens were cleared in lactic acid and mounted in Marc André II solution. The head, furca and legs were cut off from the trunk and mounted separately for detailed observation. The slide-mounted specimens were studied using a Leica DMLB microscope.

The pattern of dorsal cephalic chaetotaxy used here is modified from that Wang et al. (2013): the two posterior macrochaetae placed in the postocular area by these authors (Figure 2F in Wang et al. 2013) are here considered to belong instead to the posterior area. We follow Fjellberg (2007) for maxilla lamellae numbering and Christiansen (1964) for macrochaetotaxy. The dental spine formula follows that of Folsom (1913), in which the dental spines are arranged from basal to distal, with a slash indicating the separation between basal and medial subsegments and the Roman numerals referring to spines that are noticeably larger.

### Abbreviations

Ant.: antennal segment; PAO: postantennal organ; Th.: thoracic segment; Abd.: abdominal segment. Institutional acronyms: NJAU, Nanjing Agricultural University, Nanjing, China; MNHN, Muséum national d’Histoire naturelle, Paris, France.

## Taxonomy

### Tomocerus postantennalis sp. n.

http://zoobank.org/D2015E75-AF28-4A47-9FC2-7AC6D0568FD4

http://species-id.net/wiki/Tomocerus_postantennalis

Figs 1 – 18

#### Type locality.

Vietnam, Tuyen Quang Province: Na Hang, Khuoi Sung, Hang Khuoi Sung, in cave, 22.5013°N, 105.3649°E, 24 Dec. 2003, Louis Deharveng and Anne Bedos leg.

#### Type specimens.

Holotype female and three paratype females on slides, labelled with collectors’ sample number Vn0312-56. Deposited in MNHN (holotype and two paratypes) and NJAU (one paratype).

#### Description.

**Figure 1.**
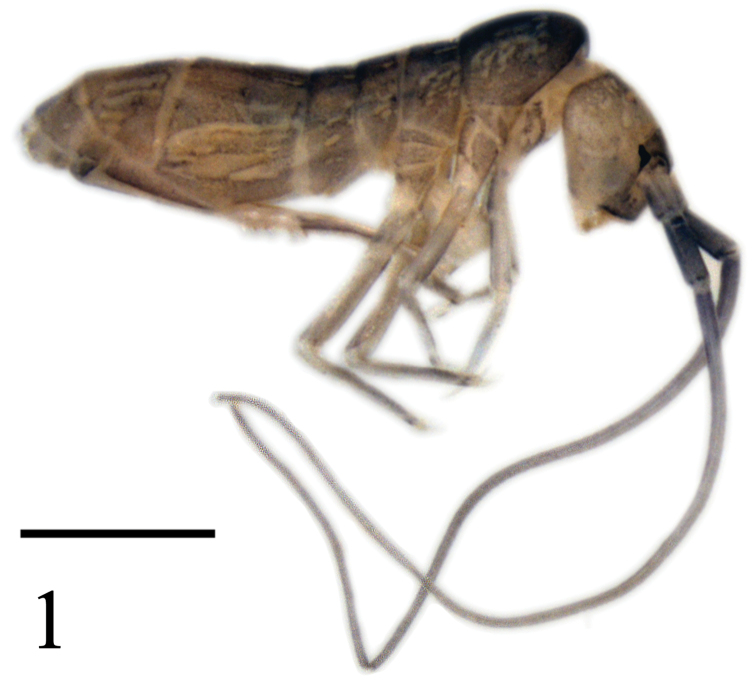
*Tomocerus postantennalis* sp. n. Appearance in alcohol. Scale bar: 1000 μm.

Body length 3.4–4.3 mm. Body with diffuse dark pigment all over; Ant. II, base of Ant. III and ventral side of Ant. I darker than other parts of antenna; eye patches black and small; clypeus, antero-dorsal region and posterior margin of head darker than other parts of head; anterior half of trunk darker than posterior half; head and trunk with bilaterally symmetrical white pattern formed by numerous unpigmented patches (Fig. 1).

**Figures 2–11.**
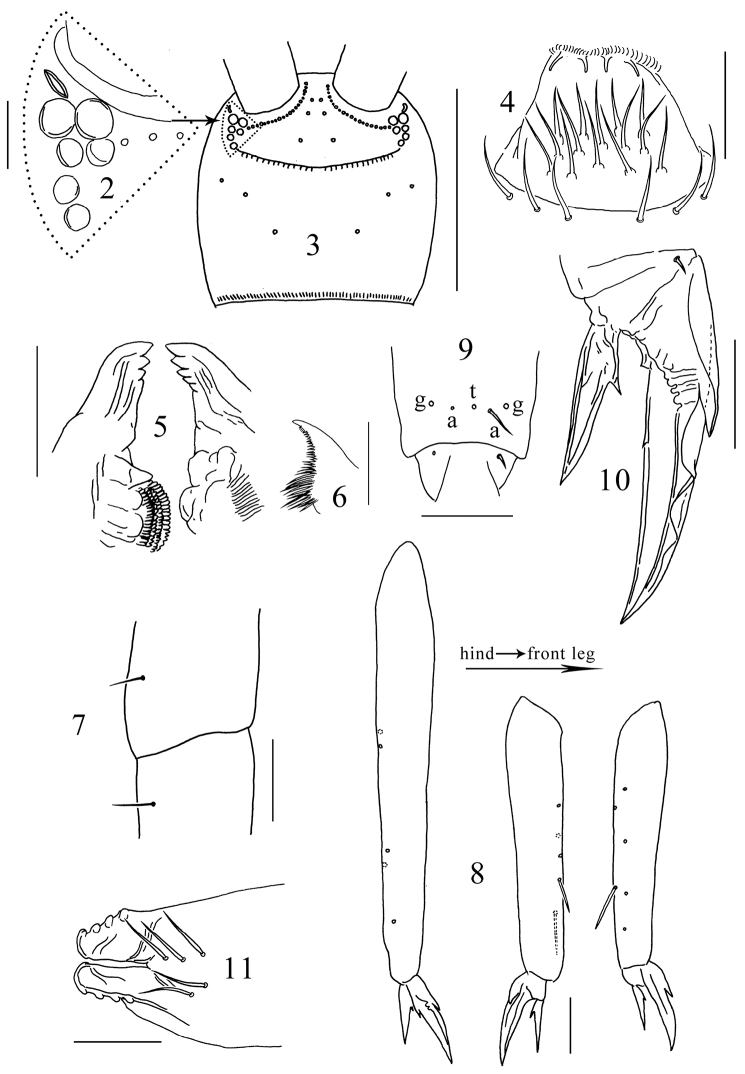
*Tomocerus postantennalis* sp. n. **2** PAO and ocelli **3** cephalic dorsal chaetotaxy **4** labrum **5** mandible **6** maxillary lamella five **7** trochanteral-femoral organ **8** tibiotarsus **9** anterior view of distal tibiotarsal chaetae (t: tenent hair, a: accessory chaetae, g: guard chaetae) **10** claw **11** tenaculum. Scale bars: **2, 7, 9, 10, 11** = 50 μm; **3** = 500 μm; **4, 5, 8** = 100 μm; **6**= 20 μm.

Antenna longer than body, Ant. IV lost, Ant. I:II:III ≈ 1.0:1.8:21.0, Ant. I and II dorsally scaled, Ant. III unscaled. Antennal chaetae poorly preserved and not studied. PAO oval, with thickened inner margin, its long axis as long as the diameter of anterior ocelli (Fig. 2). Ocelli 6+6, anterior two larger than others (Figs 2, 3). Prelabral chaetae 3+3; labral chaetotaxy 5, 5, 4, distal four chaetae stronger, anterior margin of labrum with four papillae (Fig. 4). Mandibular head asymmetrical, left mandible with four teeth, right mandible with five teeth, molar plate of left mandible distally with a cone-like tooth (Fig. 5). Basal teeth of maxillary lamella 5 prolonged, distinct beard absent (Fig. 6). Mentum (baso-lateral area of labium) with 5 smooth chaetae, other parts of labium not clearly seen. Cephalic dorsal chaetotaxy: anterior area 2, 2; interocular area 2, 0, macrochaetae absent along transverse medial line; postocular area 2+2; posterior area 1+1; posterior margin with about 30+30 short chaetae (Fig. 3). Head scaled both dorsally and ventrally.

Trochanteral-femoral organ with 1, 1 chaetae (Fig. 7); fore, middle and hind tibiotarsi ventrally with 6–7, 5, 5 spine-like chaetae (Fig. 8). Distal whorl of tibiotarsus with 11 chaetae, tenent hair thin and probably pointed (judging from its small socket), two small accessory chaetae beside tenent hair larger than pretarsal chaetae, sockets of two outer guard chaetae larger than tenent hair (Fig. 9). Unguis slender, with baso-internal ridging, two lateral teeth pointed, of moderate size; inner edge of unguis with one basal and one distal tooth, the distal tooth at about one third of the length of unguis from base. Unguiculus length 0.50–0.67 that of unguis, with one inner tooth larger than ungual teeth (Fig. 10). Scales present on all segments except pretarsus of all legs.

Ventral tube scaled both anteriorly and posteriorly, lateral flap unscaled. Each side of anterior face with ca. 50 chaetae, posterior face with ca. 90 chaetae, each lateral flap with ca. 60 chaetae; all chaetae smooth.

**Figures 12–18.**
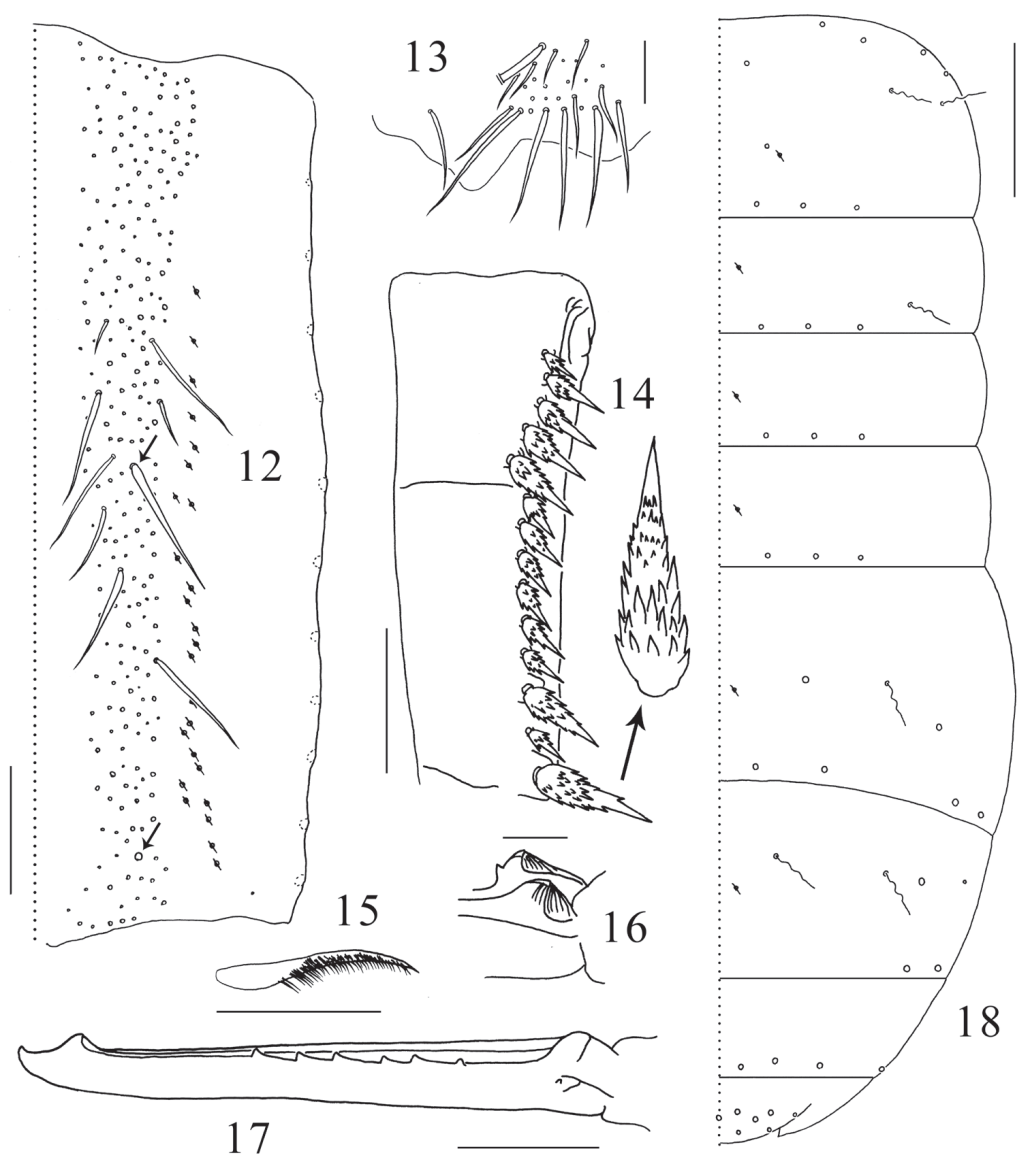
*Tomocerus postantennalis* sp. n. **12** dorsal face of manubrium (right side; prominent chaetae arrowed) **13** disto-dorsal chaetae on manubrium (left side) **14** dental spines (left side) **15** feathered chaeta on dens **16** basal teeth of left mucro **17** right mucro **18** body chaetotaxy. Scale bars: **12, 14** = 100μm; **13, 17** = 50 μm; **15, 16** = 21 μm; **18** = 400 μm. Large circles: macrochaetae; small circles: mesochaetae; wavy lines: bothriotricha; circles with a slash: pseudopores.

Tenaculum unscaled, with 4+4 teeth, anterior face with 5 small smooth chaetae (Fig. 11). Ratio manubrium:dens:mucro 3.3–4.0:4.8–5.4:1.0. Manubrium laterally with large scales and 11 chaetae, the proximal one chaeta small and smooth, the distal 10 chaetae enlarged and serrated; dorsal scales absent; each dorsal chaetal stripe with 250–300 smooth chaetae of different sizes, including 2+2 pointed prominent chaetae larger and straighter than other chaetae (arrowed in Fig. 12); 22–27 pseudopores on each side (Fig. 12); external corner chaetae as large as mesochaetae in the chaetal stripe (Fig. 13). Ventral side of manubrium covered only with scales. Dental spines formula 4–5/5–7, I, 1–2, I. Distal most spine strongest, about 0.1 times length of dens, sizes of the proximal spines increasing gradually from basal to distal. All spines compound, with numerous denticles of moderate size at basal half and smaller at distal half (Fig. 14). Dens internally divided into three subsegments, basally without outer strong chaetae or inner pointed scales, dorsally with ordinary smooth chaetae and a longitudinal central stripe of feathered chaetae (Fig. 15) from base of middle subsegment to apex of distal subsegment between ordinary chaetae, ventrally covered with small oval scales. Mucro elongate and multi-setaceous; both basal teeth with proximal lamellae, outer basal tooth with a toothlet (Fig. 16); apical and subapical teeth subequal; two dorsal lamellae beginning from subapical tooth, outer lamella ending in inner basal tooth, inner lamella ending freely beside inner basal tooth. Outer dorsal lamella with 5–7 subequal intermediate teeth (Fig. 17).

Macrochaetae distributed densely along anterior margin of Th. II (not drawn) and sparingly in posterior rows on terga. Th. II-Abd. V with 2,1/0,0,1,2,0 bothriotricha and 3,3/3,3,4,2,4 (3 central+1 lateral) posterior macrochaetae on each side; dorsal flap of Abd. VI with 13 macrochaetae (6+6 and 1 on middle line). Medial area of Th. II with two macrochaetae, the posterior one close to pseudopore; Abd. IV antero-laterally with one macrochaeta and one mesochaeta (Fig. 18). Most mesochaetae present at lateral margin of terga, especially from Abd. II to Abd. IV. Microchaetae uniformly distributed.

#### Etymology.

Named with reference to the presence of the postantennal organ.

#### Remarks.

*Tomocerus postantennalis* sp. n. is distinct from any other *Tomocerus* spp. in the presence of PAO and six prelabral chaetae. Besides, it is characterized by the unequal size of the ocelli, the lower number of cephalic macrochaetae and the reduction in the number of ungual inner teeth. Its compound dental spines are very similar to those of the Vietnamese species *Tomocerus ocreatus* Denis, 1948 and of the Chinese species *Tomocerus folsomi* Denis, 1929, which probably indicates a close phylogenetic relationship. The discrimination of these species is shown in Table 1.

**Table 1.** Discrimination of *Tomocerus postantennalis* sp. n., *Tomocerus ocreatus* and *Tomocerus folsomi* on the basis of the original descriptions and notes (Denis 1929, 1948).

Species	Body colour	Length of antennae	Unguis teeth	Shape of dental spines	Dental spines formula	Number of mucronal intermediate teeth
*Tomocerus folsomi*	yellowish with dark pigment along lateral margin of anterior terga	shorter than body	4–5	compound with very fine denticles	5–8/3–6, I, 1–2, I	6–7
*Tomocerus ocreatus*	pale	as long as body	5	compound with denticles of moderate size	3/3–4, II	8–9
*Tomocerus postantennalis* sp. n.	dark grey	longer than body	2	compound with denticles of moderate size	4–5/5–7, I, 1–2, I	5–7

## Discussion

Most tomocerids have four prelabral chaetae, which is also common number in other groups of Entomobryomorpha. The taxonomic significance of the prelabral chaetae in Tomocerinae was firstly discovered by Yosii (1966, 1967), who described several *Plutomurus* species with more than four such chaetae. As far as we know, tomocerids other than *Tomocerus postantennalis* sp. n. with six or eight prelabral chaetae belong to two genera: *Plutomurus* (*Plutomurus grahami* Christiansen, 1980, *Plutomurus ehimensis* Yosii, 1956, *Plutomurus kawasawai* Yosii, 1956, *Plutomurus gul* Yosii, 1966, *Plutomurus iwatensis* Yoshii, 1991, *Plutomurus ortobalaganensis* Jordana and Baquero, 2012, *Plutomurus kelasuricus* Martynova, 1969 and *Plutomurus marmorarius* Yosii, 1967) and *Lethemurus* Yosii, 1970 (*Lethemurus finitimus* Yosii, 1970). They all inhabit caves and most of them are eyeless, with the sole exception of *Plutomurus kelasuricus*, which has small ocelli. Although the function of prelabral chaetae is still unknown, the increased number of these chaetae appears to be a troglomorphic adaptation. The protruding prelabral chaetae probably provide a tactile sense in front of the mouthparts, and more prelabral chaetae may increase the sensitivity, which is important for living in darkness.

So far, only two species of Tomocerinae have the PAO developed in adults: *Tomolonus reductus* (Mills) and *Tomocerus postantennalis* sp. n. The former has the most reduced eyes of non-cave species (3+3) and lives in soil, while the latter has the maximum number of ocelli for the subfamily (6+6) and lives in a cave, where its eyes are useless because of darkness. PAO development in adult may therefore be assumed to compensate for deficient vision performance in both species. Under this assumption, we would expect PAO among other cave species of Tomocerinae, a presence difficult to detect given the inconspicuousness of the organ when present.

The PAO of *Tomocerus postantennalis* sp. n. is similar in shape to those of juvenile *Pogonognathellus longicornis* (Goto 1956) and some adult isotomids (Potapov 2001), but it differs from the compound PAO of *Tomolonus*, which is constituted of a small central vesicle and three larger tubercles (Mills 1948, Yosii 1967, Rusek 1977). Besides this structural difference, the PAO of *Tomocerus postantennalis* sp. n. is relatively larger than those of *Tomolonus reductus* and juvenile *Pogonognathellus longicornis*. The morphological differences in PAO between *Tomocerus postantennalis* sp. n. and *Tomolonus reductus* may reflect a functional difference or represent two evolutionary alternatives—enlargement of a simple organ or complication of a small organ, to enhance a similar function. In any case, the different PAOs in the two species are likely to be adaptive convergences rather than synapomorphies, since they are distantly related based on strong morphological differences.

Despite the presence of PAO, *Tomolonus* is more closely related to *Plutomurus*, since both have two posterior macrochaetae on the thoracic segments (Christiansen 1964, 1980, Jordana et al. 2012), a well developed trochanteral-femoral organ, strong outer basal chaetae on the dens and simple dental spines. The new species fits *Tomocerus* better than other genera because it has three posterior macrochaetae on the thoracic segments, poorly developed trochanteral-femoral organ, no strong dental chaetae and a toothlet on the outer basal tooth of the mucro.

The genus *Tomocerus* Nicolet, 1841 is so far poorly defined by a single character: the presence of a toothlet on the outer basal mucronal tooth, thus all species conforming to this criterion and lacking the diagnostic character of other genera have been assigned to this genus, resulting in a wider range of intrageneric diversity than in other genera. For instance, all types of dental spines from simple to strongly furcated can be found in *Tomocerus*, whereas in *Pogonognathellus*, *Plutomurus* and *Monodontocerus* the shape of dental spines are constant within genus. On the other hand, the single generic character is not exclusive for *Tomocerus* since *Aphaenomurus interpositus denticulatus* Yosii, 1956 and *Plutomurus vigintiferispina* Lee, 1974 also have a toothlet on the mucronal outer basal tooth. The situation of *Tomocerus* will remain problematic until a comprehensive and detail investigate is carried out.

## Supplementary Material

XML Treatment for Tomocerus postantennalis
